# Cryptic amyloidogenic elements in mutant NEFH causing Charcot-Marie-Tooth 2 trigger aggresome formation and neuronal death

**DOI:** 10.1186/s40478-017-0457-1

**Published:** 2017-07-14

**Authors:** Arnaud Jacquier, Cécile Delorme, Edwige Belotti, Raoul Juntas-Morales, Guilhem Solé, Odile Dubourg, Marianne Giroux, Claude-Alain Maurage, Valérie Castellani, Adriana Rebelo, Alexander Abrams, Stephan Züchner, Tanya Stojkovic, Laurent Schaeffer, Philippe Latour

**Affiliations:** 1 Institut NeuroMyoGène, Université Lyon1 - CNRS UMR 5310 - INSERM U1217, Lyon, France; 2 Unité fonctionnelle de neurogénétique moléculaire, CHU de Lyon - HCL groupement Est, Bron, France; 3 Département de Neurologie, Hôpital Pitié-Salpêtrière, Paris, France; 4 Clinique du motoneurone et pathologies neuromusculaires, CHRU de Montpellier, Montpellier, France; 5 Centre de références des maladies neuromusculaires, CHU de Bordeaux, Bordeaux, France; 6 Centre de référence des maladies neuromusculaires, Hôpital Pitié-Salpêtrière, Paris, France; 7 Service de Neurologie, CH de Valenciennes, Valenciennes, France; 8 Institut de pathologie, CHU de Lille, Lille, France; 9 Dr John T. MacDonald Foundation Department of Human Genetics, Institute of Human Genomics, University of Miami, Miller School of Medicine, Miami, USA; 10 Institut de Myologie, Hôpital Pitié-Salpêtrière, 47-83 boulevard de l’Hôpital, 75013 Paris, France; 11 Centre de Biotechnologie Cellulaire, CBC Biotec, CHU de Lyon - HCL groupement Est, Bron, France

## Abstract

Neurofilament heavy chain (*NEFH*) gene was recently identified to cause autosomal dominant axonal Charcot-Marie-Tooth disease (CMT2cc). However, the clinical spectrum of this condition and the physio-pathological pathway remain to be delineated. We report 12 patients from two French families with axonal dominantly inherited form of CMT caused by two new mutations in the *NEFH* gene. A remarkable feature was the early involvement of proximal muscles of the lower limbs associated with pyramidal signs in some patients. Nerve conduction velocity studies indicated a predominantly motor axonal neuropathy. Unique deletions of two nucleotides causing frameshifts near the end of the *NEFH* coding sequence were identified: in family 1, c.3008_3009del (p.Lys1003Argfs*59), and in family 2 c.3043_3044del (p.Lys1015Glyfs*47). Both frameshifts lead to 40 additional amino acids translation encoding a cryptic amyloidogenic element. Consistently, we show that these mutations cause protein aggregation which are recognised by the autophagic pathway in motoneurons and triggered caspase 3 activation leading to apoptosis in neuroblastoma cells. Using electroporation of chick embryo spinal cord, we confirm that NEFH mutants form aggregates in vivo and trigger apoptosis of spinal cord neurons. Thus, our results provide a physiological explanation for the overlap between CMT and amyotrophic lateral sclerosis (ALS) clinical features in affected patients.

**Electronic supplementary material:**

The online version of this article (doi:10.1186/s40478-017-0457-1) contains supplementary material, which is available to authorized users.

## Introduction

Charcot-Marie-Tooth disease (CMT) refers to a heterogeneous group of chronic inherited motor and sensory disorders of the peripheral nervous system. CMT are classified according to their axonal or demyelinating feature on nerve conduction studies, and their mode of inheritance [10]. The autosomal dominant axonal forms are termed CMT2. The number of genes associated with CMT is progressively expanding, particularly since the development of next-generation sequencing. Several of these genes are expressed in both the central and peripheral nervous system, such as neurofilaments, which have been implicated in several neurodegenerative diseases, including ALS [24]. Neurofilaments are intermediate filaments exclusively expressed in neurons in the central and peripheral nervous system. They have important cytoskeletal functions such as the regulation of axonal growth and diameter [15]. Neurofilaments are composed of three subunits defined by their molecular weight: NEFL (light), NEFM (medium), and NEFH (heavy) [23, 24], encoded by *NEFL*, *NEFM* and *NEFH* genes, respectively. Mutations in *NEFL* are known to cause both axonal and demyelinating forms of CMT and manifest with various clinical phenotypes, sometimes with additional pyramidal signs [2, 4, 13, 19, 25]. Mutations in the *NEFH* gene have been suggested to play a role in the pathogenesis of sporadic amyotrophic lateral sclerosis (ALS), but with conflicting results [28].

Recently, *NEFH* mutations have been identified as a rare cause of autosomal dominant CMT, with two families reported to date [27]. The clinical and electrophysiological phenotype in these two families was characterized by a severe, predominantly motor, axonal neuropathy, with significant walking difficulties in early adulthood. Similar to our families, the two mutations (c.3010_3011delGA and c.3017_3020dup) cause the loss of the stop codon and the translation of 40 additional amino acids which encode a cryptic amyloidogenic element (CAE) and cause protein aggregation [27].

Here, we report two French families presenting with an axonal, dominantly inherited form of CMT characterized by prominent motor deficit affecting both the distal and proximal muscles, and signs of central nervous system involvement, caused by two previously unreported mutations in the *NEFH* gene. We show that those new mutations cause protein aggregation, not only in neuroblastoma cells as similar mutations previously reported, but also in primary mouse motoneurons. We further show that this type of mutations also induces neuronal apoptosis, both in neuroblastoma cells and in vivo in spinal cord neurons using in ovo chick spinal cord electroporation. Our results thus provide a physiological basis to the pathogenicity of *NEFH* mutations that interfere with neurofilament assembly via protein sequestration and cause neurotoxicity, which explains the overlapping clinical features of *NEFH* mutations with those of motor neuron disease.

## Materials and methods

### Patients

**Fig. 1:**
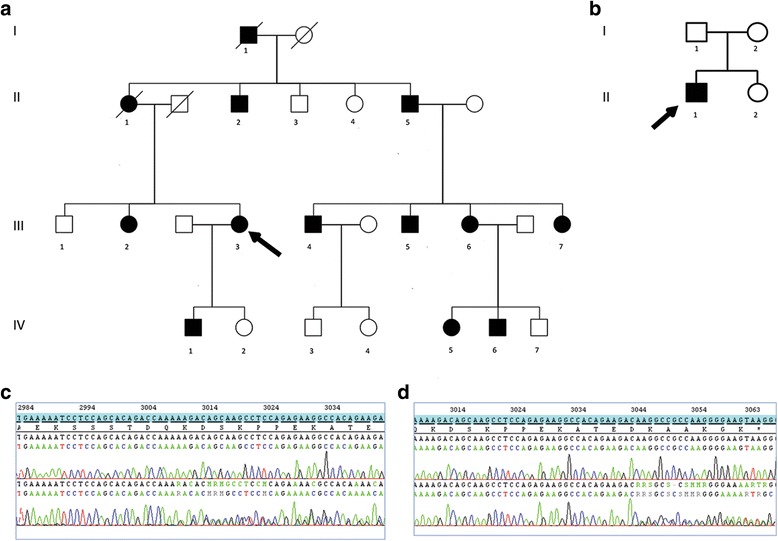
Pedigree of the two families. **a** Family 1. **b** Family 2. *Arrow* indicates the proband. *Slash lines* indicate dead individuals. *Squares* are males and *circles* are females. *Filled symbols* represent affected subjects and *empty symbols* unaffected subjects. **c** Family 1 - *NEFH* C-terminal sequence showing nucleotides 2982 to 3041 (reference transcript NM_021076.3). *Top*: control sequence. *Bottom*: frameshift mutation c.3008_3009del (p.Lys1003Argfs*59). **d** Family 2 - *NEFH* C-terminal sequence from nucleotides 3006 to the stop codon of the normal transcript (reference transcript NM_021076.3) *Top*: control sequence. *Bottom*: frameshift mutation c.3043_3044del (p.Lys1015Glyfs*47)

The patients were identified as part of our on-going genetic studies in CMT. Patients were all of French ascendance. Patients were recruited, enrolled and sampled according to the protocols of the institutional review board at the Pitié-Salpêtrière Hospital. Written informed consent was obtained for participation in the study. Patients displayed a clinical and electrical phenotype of axonal motor and sensory neuropathy, with no mutations in known CMT2 genes at that time. Twelve patients belonging to two different families (Fig. 1) were included in the study.

### Clinical assessment

Patients were seen in neuromuscular centres and assessed by senior neurologists specialized in neuromuscular disorders in Paris (TS and OD), Lille (TS), Bordeaux (GS), Valenciennes (MG) and Montpellier (RJM). Clinical assessment included medical history and neurological examination.

### Neurophysiological study

Electrodiagnostic studies included nerve conduction studies in the upper and lower limbs, and electromyography (EMG) using concentric needle electrodes in at least three muscles. Electrodiagnostic studies were performed using conventional equipment and standard methods. Skin temperature was maintained in the range of 32° to 34 °C. Patients were classified as having axonal neuropathy if they had a nerve conduction velocity in the median nerve above 38 m/s [12].

### Histological study

A nerve biopsy (superficial peroneal sensory nerve) was performed in patients II-1, III-4 and III-7 of family 1. A muscle biopsy (tibialis anterior) was performed in patients III-3, III-4 and III-7 of family 1 and patient II-1 of family 2. Muscle and nerve biopsies were processed and assessed according to standard techniques [33].

### Molecular analysis

Mutations in genes frequently associated with axonal CMT (*MFN2*, *GJB1*; *MPZ*, *TRPV4, NEFL*, *GDAP)* and amyotrophic lateral sclerosis (ALS), such as *SOD1*gene, were first excluded by Sanger sequencing. In family 1, four patients were then studied by sequencing a panel of 4813 genes associated with known clinical phenotypes (Illumina® TruSight One Sequencing Panel) on Illumina® NextSeq500 sequencer. In family 2, the index case and his two parents were sequenced by the same method. Confirmation of the putative deleterious variants in *NEFH* and familial studies were done by Sanger sequencing.

### Plasmid construct and mutagenesis

The construct encoding for the human NEFH was a gift from Dr Sidransky [21] and it was subcloned in a pCAGEN eGFP C1 plasmid allowing optimal expression in neurons. NEFH N-terminus end was fused with monomeric eGFP (derived from peGFP-C1, clonetech) using Gibson assembly kit (E5510, NEB) following the manufacturer recommendation to obtain pCAGEN eGFP NEFH referred to as WT NEFH thereafter. The mutant forms of *NEFH*, pCAGEN eGFP-NEFH plasmid were obtained by site-directed mutagenesis using QuickChange II XL Mutagenesis Kit (from Agilent technology #200521). Mutant *NEFH* plasmids harboring c.3008_3009delAA, c.3043_3044delAA and c.3010_3011delGA are referred respectively as c3008, c3043 and c3010 in the text. Untagged NEFH plasmids (WT and CAE, c.3010_3011delGA mutation) and eGFP-NEFL plasmids were provided by Pr Zuchner (University of Miami, USA). Plasmids prepared with the EndoFree Maxiprep Kit (Macherey-Nagel, Düren, Germany) were routinely diluted at 2 μg/μl.

### Cell line culture and transfection

Human neuroblastoma cell line SH-EP [3, 6] were grown on 12 mm coverslips in Dulbecco’s Modified Eagle Medium (DMEM, gibco) media supplemented with 10% fetal bovine serum (FBS, Gibco) and 1% penicillin/streptomycin (Gibco) and transfected at 60% of confluence with jetPRIME (Polyplus-transfection) according to the manufacturer’s protocol. 1, 2 or 3 days after transfection, cells were fixed with 4% paraformaldehyde for 20 min, and washed with PBS.

### Primary motoneuron culture and transfection

Spinal cord motoneurons were prepared from E12.5 OF1 mice embryos as described by Henderson et al. [14] with minor modifications. Briefly, anterior horn of the embryo were dissected in HBSS supplemented with 4.5 g/l glucose and 7 mM HEPES (invitrogen). Motoneurons were purified by using a 6% OptiPrep density gradient medium (D1556, Sigma). Then, motoneurons were resuspended in supplemented Neurobasal medium (Invitrogen) containing 1 ng/ml brain-derived neurotrophic factor (Peprotech), 1 ng/ml glial cell line–derived neurotrophic factor (Peprotech), and 10 ng/ml ciliary neurotrophic factor (Peprotech) and were seeded on polyornithin/laminin-coated glass coverslips (P8638 and L2020, Sigma). After two days in vitro, plasmid transfections were done by Magnetofection following the manufacturer recommendations (OZBiosciences). Two or four days later, motoneurons were fixed using 4% paraformaldehyde for 20 min, and washed with PBS.

### Embryonic chick spinal cord electroporation

In ovo electroporation of chick embryos (*Gallus gallus*; EARL Morizeau, Dangers, France) was performed as previously described [26]. Briefly, the constructs were introduced into the lumen of neural tube at the caudal level in stage HH14–15 embryos [11]. Stage HH24–26 chick embryos were harvested and isolated in sterile phosphate-buffered saline (PBS) and defined fragments of the neural folds, where the eGFP expression was observed, were dissected, fixed in 4% paraformaldehyde and embedded in 7,5% gelatin/15% sucrose, and frozen at −40°c in isopentane. Twenty μm frozen tissue sections were performed for immunostaining analysis.

### Immunocytochemistry

Fixed cells or frozen tissue sections were blocked and permeabilized with PBS containing 4% bovine serum albumin, 2% goat serum, 100 mM glycine and 0.3% Triton X-100. Primary antibody was applied overnight at 4 °C diluted in blocking solution, washed 3 times in PBS, incubated for 2 h in the secondary antibody at room temperature, with DAPI, then washed four times in PBS, mounted in Vectashield® and imaged with confocal microscope Zeiss LSM800 or imaged with conventional microscope Zeiss Axiovert 135 M equipped with the camera Leica DC 350 FX CCD monochrome. The following antibodies were used: anti-acetylated tubulin (1/400, clone 6-11B-1, Sigma), anti p62 (1/200, GP62-C, Progen, Germany), anti-Ubiquitin conjugated protein1 (1/100, BML-PW8810, Enzo), anti - Lc3b (1/100, #2775, Cell Signaling Technology, Danvers), anti-beta3 tubulin (1/500, TUJ1, Biolengend), anti-NEFM (1/500, Poly28410, Biolegend), anti-NEFH (1/2000, Smi32, Biolegend), anti-cleaved caspase 3 (1/100; 5A1E, Cell Signaling Technology, Danvers).

### Western blot

Triton X100-soluble and -insoluble protein fractionation were performed following cell lysis in 20 mM NaCl, 20 mM Tris-HCl, pH 7.4, 5 mM MgCl2, 0.1 mM EDTA, 0.1% Triton X-100, and protease cocktail, for 30 min at 4 °C. Then, cell extracts were centrifuged for 15 min at 15000 g to separate soluble (supernatant) and insoluble (pellet) fractions. Pellet fraction was suspended in RIPA buffer. Soluble (S) and insoluble (I) fractions were resolved by SDS-PAGE and transferred on Nitrocellulose transfer membrane. Western blot was performed with anti-GFP (A11122, Thermo) and anti-GAPDH (2118 CST), followed by chemiluminescent detection using horseradish peroxidase-conjugated antibodies and the SuperSignal West Pico (Thermo Scientific) reagent.

### Statistical analysis

Each experiment was repeated at least twice. Data were analysed with Excel (Microsoft) or SigmaStat 3.5 (Systat Software Inc). Data from more than two groups each showing normality and equal variance were analyse with one way ANOVA followed by Dunnett’s test to compare several treatment group to a control group. Otherwise, data that do not showed a Gaussian distribution were analyse with one way Kruskal-Wallis test followed by Dunn posthoc test.

## Results

### Clinical phenotype

The clinical features of family 1 and 2 are summarized in Table 1.

**Table 1:** Clinical features of patients with *NEFH* mutations

Family/Patient Gender/Age (years)	Age at onset (years)	Initial symptoms	Pattern of muscle weakness				Scoliosis	Sensory involvement		Pyramidal signs	Additional clinical signs	Ambulation
			UL (prox)	UL (dist.)	LL (prox.)	LL (dist.)		UL	LL			
1 / II – 1 F/70	40	Waling difficulties	++	++	+++	+++	−	+	+	Yes Brisk achilean reflexes	No	Bedridden
1 / II – 5 M/68	38	Walking difficulties	−	+	+	++	−	−	+	No Areflexia 4 limbs	No	Ambulant without help
1 / III – 2 F/55	30	Walking difficulties	−	−	++	++	−	−	+	No Achilles tendon areflexia	No	Ambulant without help
1 / III – 3 F/51	15	Feet dysesthesia Walking difficulties at age 27	−	+	++	+++	−	−	+	No	Hypophonia	Ambulant With a walker
1 / III – 4 M/50	30	Walking difficulties	−	++	+	++	−	−	+ Vibration hypoesthesia	No	No	Ambulant without help
I / III - 5 M/49	43	Walking difficulties	−	+	+	+	−	−	+	No Achilles tendon areflexia	No	Ambulant without help
1 / III - 6 F/46	40	Walking difficulties	−	−	++	+	−	−	No	No LL areflexia	No	Ambulant without help
1 / III - 7 F/44	34	Walking difficulties	−	−	++	+++	−	−	Distal pinprick hypoesthesia	Yes Spread of patellar reflexes	No	Ambulant with one can
1 / IV - 1 M/23	15	Running difficulties	−	−	−	+	−	−	−	Yes Brisk reflexes in 4 limbs	No	Ambulant without help
1 / IV – 5 F/23	−	Asymptomatic	−	−	−	−	−	−	−	No LL areflexia	No	Ambulant
1 / IV - 6 M/17	−	Asymptomatic	−	−	−	−	−	−	−	No Achilles tendon areflexia	No	Ambulant
2 / II - 1 M/23	5	Walking difficulties	+	+	++	+++	+	−	+	No Areflexia	Gastroparesis	Ambulant without help

M = male. F = female. Y = years. - = absent. + = mild. ++ = moderate. +++ = severe. UL = upper limb. LL = lower limb. Prox = proximal. Dist = distal

#### Family 1

The affected siblings were born from non-consanguineous parents (Fig. 1a). The family displayed an autosomal dominant inheritance pattern. The propositus (III-3) was a 49-year-old woman. She had normal developmental milestones and no walking or running difficulties in childhood. At age 27, she developed difficulties climbing stairs. Muscle weakness worsened gradually and involved both distal and proximal muscles in the lower limbs, with difficulties arising from squat position. At 30 years of age, she developed proximal weakness in the lower limbs, involving particularly the iliopsoas muscle (MRC score 3/5), in addition to the distal weakness. At age 49, she was able to walk around 50 m with a walker, and used a wheelchair for longer distances. She was still able to climb stairs. Romberg’s test showed mild postural instability. Heel or tiptoe walking was impossible. Squatting was impossible. Gower’s manoeuvre was positive. Muscle strength examination showed a severe motor deficit in both proximal and distal muscles of the lower limbs (tibialis anterior 1/5, peroneus longus and tibialis posterior muscles MRC 2/5 both sides; quadriceps 3/5 right side, 1/5 left side; hamstring muscles 2/5 both sides; gluteus medius 3/5 both sides; iliopsoas 2/5 both sides). There was distal motor deficit in the upper limbs (abductor digiti minimi 3/5 right side, 3+/5 left side; normal strength in the other muscles). There were no fasciculations. There was distal and proximal muscle wasting in the lower limbs, and distal muscle wasting in the upper limbs. She had pes cavus. Deep tendon reflexes were absent and plantar reflex was flexor. She had distal hypoesthesia in the lower limbs (pin, touch and vibration). She reported frequent episodes of hypophonia, suggestive of associated vocal cord involvement. Cranial nerve examination was otherwise normal.

Other affected family members had normal milestones and could walk at normal ages (Table 1). Two of them had difficulties running and performing in gymnastics (III-3 and IV-1). Age of onset was around 50 years in generation I, 40 years in generation II, 30 years in generation III and 15 years in generation IV. Patients of the fourth generation had neither any symptoms (IV5 and IV6) nor reported running difficulties in childhood (IV1). On examination, they have mild symptoms such as mild distal lower limbs weakness, brisk or absent of lower limb deep tendon reflexes. They have been diagnosed early in their life since they were aware of symptoms related to the neuropathy in the family. Even mild neurological symptoms led their parents to seek for a neurological and neurophysiological exam confirming the neuropathy. Inaugural signs in most patients were progressive, distal lower-limbs muscles weakness and stepping gait. They progressively developed muscle wasting in the legs. Weakness and wasting in the proximal muscles of the lower limbs appeared within the third decade of the disease, and caused waddling gait. Motor deficit was clearly prominent in the iliopsoas whereas the quadriceps and hamstring muscles were better preserved during the third decade. However, the weakness spread over years to all proximal muscles in the lower and to distal muscles of the upper limbs. Indeed, the propositus's mother (patient II-1) presented at the age of 70 with diffuse motor weakness involving the distal and proximal muscles of the four limbs, associated with brisk reflexes. Most patients had cramps. Pes cavus was a consistent feature. Deep tendon reflexes absent in most patients, but three of them (II-1, III-7, IV-1) had brisk patellar reflexes. There was no Babinski or Hoffman sign. There were mild sensory abnormalities in all patients, with progressively ascending superficial and deep sensory alterations. The disease evolved progressively and most patients used a wheelchair around 50 years. There were no associated features such as visual loss, deafness, cranial nerve abnormalities, cerebellar syndrome, seizures or cognitive disturbances. One of the patients (III-4 of family 1) experienced a malignant hyperthermia following a surgical procedure.

#### Family 2

The propositus (II-1) was a 23-year-old man born from non-consanguineous asymptomatic parents (Fig. 1b). There was no familial history of neuropathy. He was born at term of an uncomplicated pregnancy. In early infancy, he presented with walking clumsiness with frequent falls and difficulties jumping. A pectum excavatum was noted. He had waddling gait with difficulties climbing stairs. Progressively, he developed drop foot gait and could not walk on heels. He had several episodes of ankle sprains. Since age 16, he developed progressive atrophy in the lower limbs muscles and complained of frequent cramps. At age 23, he could walk approximately one kilometre without help. He had waddling gait and bilateral foot drop. Heel or tiptoe walking and squatting were impossible. Gower’s manoeuvre was positive. He had mild postural instability at Romberg’s test. Muscle strength examination revealed a symmetric motor deficit in distal and proximal lower limbs (MRC score: tibialis anterior 1+/5; soleus 1/5, peroneus longus 0/5, hamstring and gluteus medius 4/5, quadriceps and psoas 3/5). Upper limbs examination showed symmetric proximal and distal deficit (distal hand muscles, biceps brachii and triceps brachii 4/5 and deltoid 3/5). There was no axial deficit. There was severe muscle wasting in the lower limbs, distally and proximally, and Achilles tendons contractures. There were no fasciculations. He had bilateral scapular winging and pes cavus. Deep tendon reflexes were weak, plantar responses were flexor. He had distal hypoesthesia in the lower limbs (pin, touch and vibration). Neurological examination was otherwise normal. He had no associated features except for gastroparesia, with episodes of morning vomiting.

### Electrophysiological findings

Nerve-conduction velocity studies are shown in Table 2 for families 1 and 2. There was evidence of a motor and sensory axonal neuropathy predominantly affecting the lower limbs. EMG showed neurogenic changes in distal muscles in all patients and in proximal muscles in the most severely affected patients). Two patients (IV - 5 and IV - 6) in family 1 underwent an electrodiagnostic study before the genetic investigation at ages 23 and 17 years, respectively, which displayed a sensorimotor axonal neuropathy.

**Table 2:** Electrophysiological findings

	Motor nerve conduction						Sensory nerve conduction		
	Median nerve		Ulnar nerve		Peroneal nerve		Median nerve	Ulnar nerve	Sural nerve
Family/Patient	Amp (mV)	CV (m/s)	Amp (mV)	CV (m/s)	Amp (mV)	CV (m/s)	Amp (μV)	Amp (μV)	Amp (μV)
1/II – 5	12.1	53.8	NA	NA	0.12	31.1	NO	NO	NO
1/III – 2	7.4	54.3	NA	NA	5	43.2	13.7	NA	4.5
1/III - 3	10.6	65	7.6	52	2.4	31	12	5	2
1/III – 4	8.2	50	NA	NA	NA	NA	NO	NO	NO
1/III – 5	6.4	45.1	8.5	49.8	NO	NO	NO	NO	NO
1/III – 6	7.8	44.2	11.4	50	2.4	30.8	6.3	3.3	NO
1/III – 7	3.4	51.1	9	43.6	4.1	41.1	4.2	7.7	4.5
1/IV – 1	13.07	53	11.81	56	0.77	37	47.8	19.4	9.6
1/IV – 5	8.2	55.2	NA	NA	5.8	39.1	10.1	10.3	NO
1/IV – 6	8.0	50.2	NA	NA	6.5	38.5	11.5	14.7	4.1
2/II – 1	5.77	54	6.8	59	1.35	34	3.5	NO	NO

The right side of the nerves is represented in this table. *Amp* amplitude. *CV* conduction velocity. *NO* Not obtained. *NA* Not available

### Histological findings

Nerve biopsy of patient II-1 of family 1 showed signs of chronic denervation with no inflammatory infiltrates or vascular abnormalities. There was evidence of metachromatic staining of the Schwann cells. Muscle biopsy of patient III-3 of family 1 showed signs of chronic denervation associated with reinnervation. Some mitochondrial abnormalities were observed with mitochondrial loading in some fibers and three muscle fibers were Cox negative. Muscle biopsy of patient III-4 of family 1 showed muscle fiber atrophy with signs of chronic denervation and reinnervation. Cox staining was normal. Nerve biopsy of the same patient showed the rarefaction of large myelinated fibers and some fibers with thin myelin sheath (Additional file 1: Figure S1). There were no signs of inflammatory deposits or Congo red staining. Muscle biopsy of patient III-7 of family 1 showed muscle atrophy with signs of denervation following a fascicular distribution. Muscle biopsy of patient II-1 of family 2 showed atrophy and grouping of muscle fibers suggestive of neurogenic pattern. There were no mitochondrial abnormalities. Cox staining was normal.

### Molecular analysis

In family 1, only one *NEFH* variant was shared by the four patients, and absent in ExAC database. In family 2, 30 variants absent in ExAC database were found for the index case. The variant in *NEFH* was the only one absent in his two parents. Both variants were novel deletions of 2 nucleotides in the extreme C-terminus of *NEFH* gene: in family 1, c.3008_3009del (p.Lys1003Argfs*59), and in family 2 c.3043_3044del (p.Lys1015Glyfs*47). The segregation of the mutation in family 1 was confirmed in 13 at-risk subjects (11 affected, 2 no affected). The mutation occurred de novo in family 2.

### Mutant NEFH forms aggresomes in human neuroblastoma cells line

**Fig. 2:**
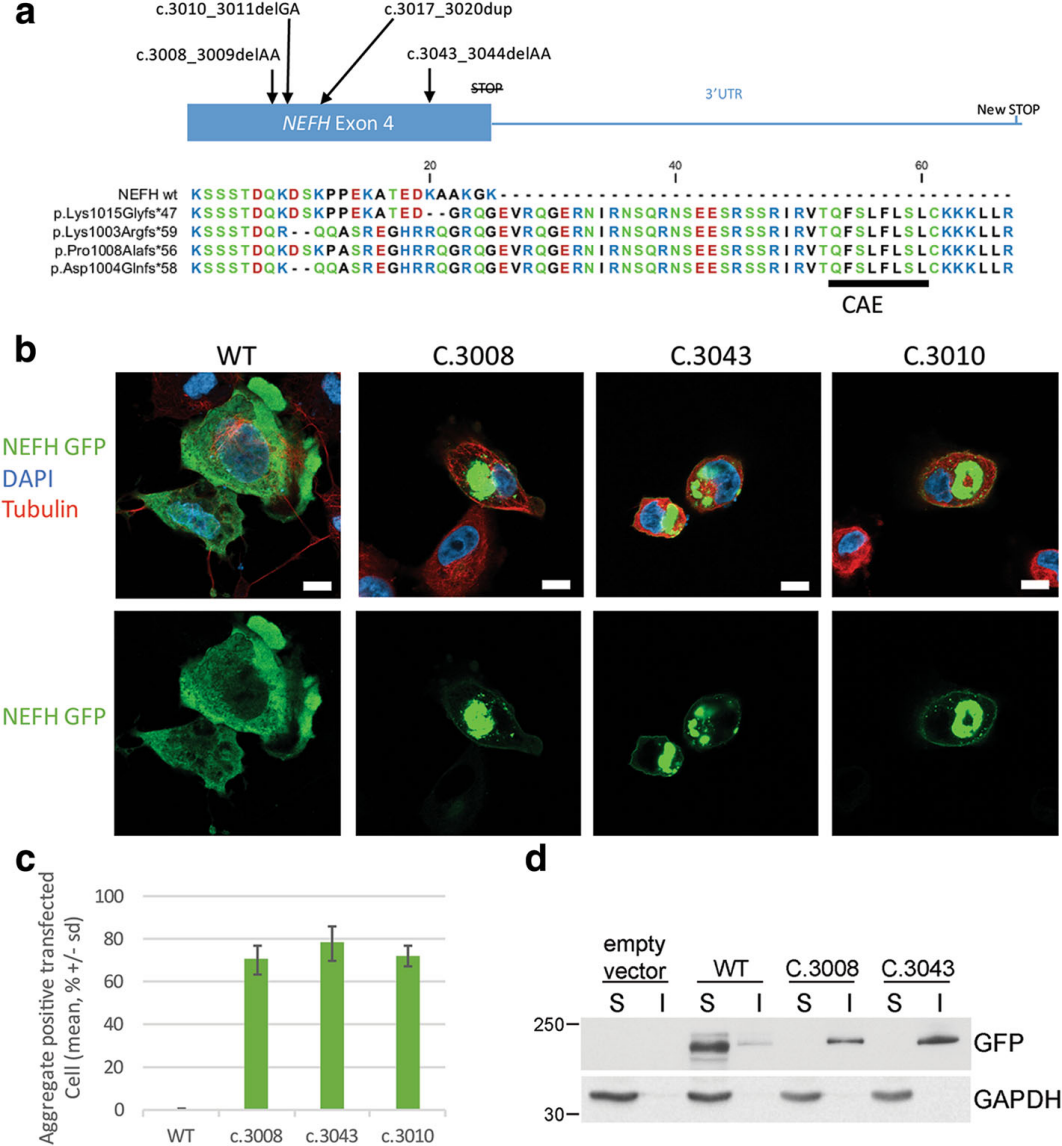
NEFH mutations cause protein aggregation in SH-EP. **a** Amino acid alignment of normal and mutant *NEFH* C-terminal parts reported in the CMT2cc presenting the Cryptic Amyloidogenic Element translation and their differences. Amino acid color correlates with polarity: hydrophobic in *black*, hydrophilic in *green*, acidic in *red* and basic in *blue*. **b** Confocal images of SH-EP transfected with eGFP tagged NEFH vectors and counterstained for tubulin in *red* and nucleus (DAPI) in *blue*. *Scale bar*: 20 μm. **c** Quantification of SH-EP cells with protein aggregates. Values represent mean +/− standard deviation of 15 fields, repeated in triplicates and analyzed by one-way ANOVA on ranks followed by Dunn posthoc test (*P* < 0.001). **d** 0.1% Triton X-100 Soluble and Insoluble fractionation revealed by western-blotting using GFP antibody to stain NEFH fusion protein and GAPDH gene to normalize

Both mutations cause the loss of the termination codon, leading to the translation of 40 additional amino acids until the next stop codon at the 3’UTR, as described in Rebelo et al. (c.3010; Fig. 2a). We thus investigated whether our mutations could cause protein aggregation. Expression vectors for eGFP tagged NEFH were transfected in the human neuroblastoma cell line SH-EP. Twenty-four hours after transfection all mutant forms (c.3008, c.3043 and c.3010) formed aggregates visible at 10× lens objective in more than 70% of the transfected cells, without significant difference between c.3008 (70.6% +/− 6.7), c.3043 (78.3 +/− 8.0) or c.3010 (72.3% +/− 4.9) (Fig. 2c). Conversely, WT NEFH formed aggregates in less than 1% of the transfected cells (0.3% +/− 0.6). This result was consistent with the one obtained by Rebelo et al.

**Fig. 3:**
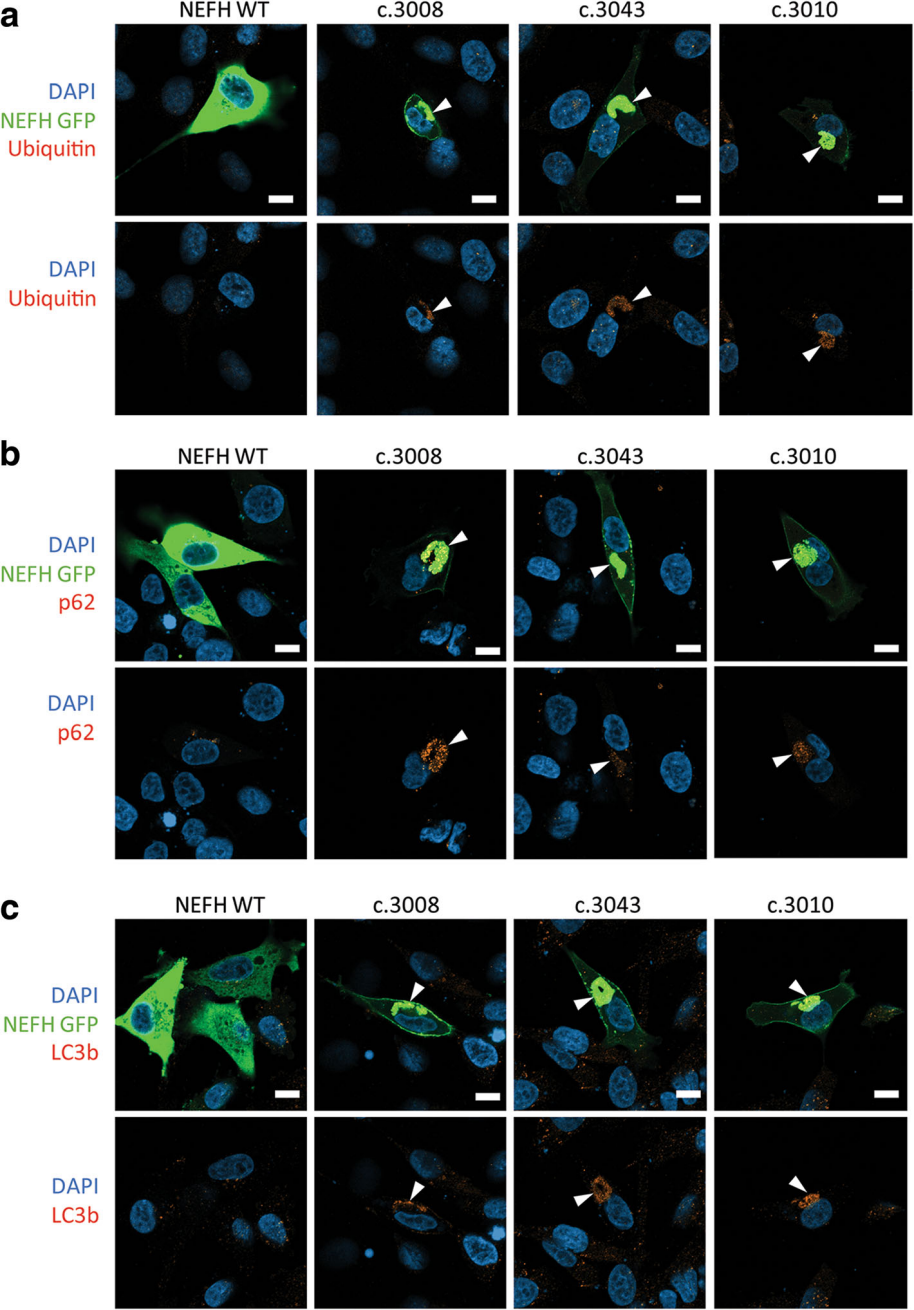
NEFH aggregates form aggresomes. eGFP tagged NEFH proteins form perinuclear aggregates called aggresome containing ubiquitin, p62/SQSTM1 and LC3b. Confocal images of transfected SH-EP counterstained for nucleus with DAPI in *blue* and ubiquitin conjugated protein1 (**a**), p62/SQSTM1 (**b**), and LC3b (**c**) in *red*. Scale bar: 20 μm

Next we investigate the subcellular distribution of mutant NEFH proteins, forty-eight hours after transfection. At low expression levels, WT NEFH incorporated into a filamentous network in the cytoplasm (Additional file 2: Figure S2). At higher plasmid concentration, overexpressed WT NEFH was distributed homogenously in the cytoplasm as revealed by the co-staining with acetylated tubulin and DAPI (Fig. 2b). Conversely, mutant NEFH proteins was always found under the plasma membrane and was also either localized in small aggregates scattered over the cytoplasm or accumulated in a single prominent perinuclear aggregate next to the microtubule organization center (MTOC). Such prominent perinuclear inclusion bodies formed next to the MTOC are commonly named aggresomes [18]. Accumulation of insoluble proteins is characteristic of aggresomes. Solubility in Triton X-100 was used to evaluate the solubility of NEFH proteins expressed in SH-EP cells. As shown in Fig. 2d, WT NEFH was solubilized by 0.1% Triton, whereas a significant proportion of mutant NEFH proteins were insoluble. Aggresomes are also characterized by accumulation of ubiquitin conjugates. As expected, aggregates of mutant NEFH proteins colocalized with mono- and poly-ubiquitinated conjugates as evidenced by staining of ubiquitin conjugated (Fig. 3a).

### Mutant NEFH proteins are addressed to the autophagic pathway

Aggresome formation has been proposed to allow isolation of toxic misfolded proteins and to act as key step for the disposal of protein aggregates by autophagy. In addition, the autophagy role in motoneuron and muscle disorders associated with protein aggregates is well known [5, 7, 29]. We thus investigated the distribution of the protein p62/SQSTM that recruits poly ubiquitinated substrates to autophagy. Immunofluorescence experiments revealed that p62 colocalized with aggresomes containing mutant NEFH (Fig. 3b). Finally, we examined the distribution of LC3b, an autophagosome marker. Consistently with p62 localization, we found that LC3b accumulated with aggresomes containing mutant NEFH proteins (Fig. 3c). Altogether, these observations suggest that the cells sequester mutant NEFH proteins in aggresomes from where they direct them to autophagy for degradation.

### NEFH mutants perturb the neurofilament network in vitro

**Fig. 4:**
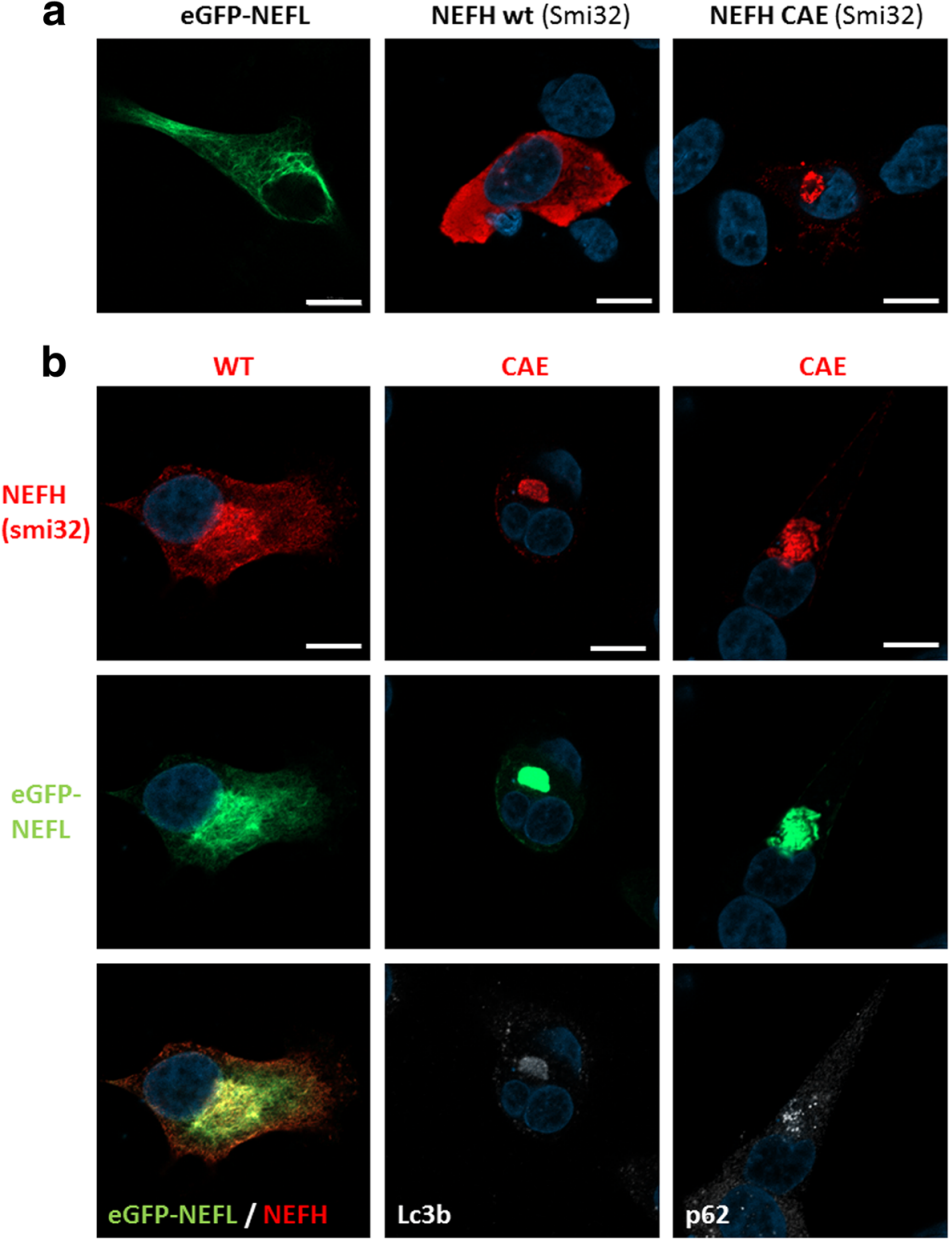
NEFH mutation destabilised NEFL filamentous network in vitro. **a** Confocal images of transfected SH-EP with eGFP-NEFL or untagged NEFH WT or untagged NEFH-CAE (c3008_3010delGA mutation). Untagged NEFH proteins were stained using anti-NEFH antibody Smi32. *Scale bar*: 10 μm. **b** Confocal images of co-transfected SH-EP with eGFP-NEFL and untagged NEFH WT or CAE. The mutant untagged NEFH-CAE proteins form aggresome containing NEFL, p62/SQSTM1 and LC3b. *Scale bar*: 10 μm

The ability of NEFH mutants to form an aggresome in the presence of a dense intermediate filamentous network containing the NEFH partner NEFL was investigated. Monomeric eGFP tagged NEFL, untagged NEFH WT or untagged NEFH CAE mutants (referring to the mutant c.3010_3011DelAG, Rebelo et al.) were overexpressed in SH-EP cells, alone or in combination. As expected, SH-EP expressing eGFP-NEFL formed a typical filamentous network. In agreement with the results presented above, WT NEFH homogenously distributed in the cytoplasm whereas NEFH CAE mutants formed perinuclear aggregates, as revealed by the Smi32 staining (Fig. 4a). As expected, eGFP-NEFL and WT NEFH colocalized in a dense filamentous network in SH-EP cells (Fig. 4b). Interestingly, eGFP-NEFL and mutant NEFH-CAE proteins colocalized restrictedly in an aggresome containing p62/SQSTM1 and LC3b (Fig. 4b). Therefore, NEFH mutants not only form aggresomes, but also interact with NEFL and destabilizes the neurofilaments network.

### Mutant NEFH proteins activate caspase 3 dependent cell death in vitro

Aggresome formation usually indicates a cellular stress response. To determine if mutant NEFH could be cytotoxic, we investigated for signs of cellular stress. NEFH is a key component of intermediate filaments that contribute to the formation of the cytoskeleton. We thus first examined the morphology of transfected cells 24 h after transfection. Metamorph analysis of the morphology of more than 1000 cells chosen stochastically demonstrated that cells expressing mutant NEFH presented a significantly reduced average radius and a significantly increased average shape factor compared to cells expressing WT NEFH (Additional file 3: Figure S3). Together, this indicated that cells expressing mutant NEFH are smaller and rounder.

**Fig. 5:**
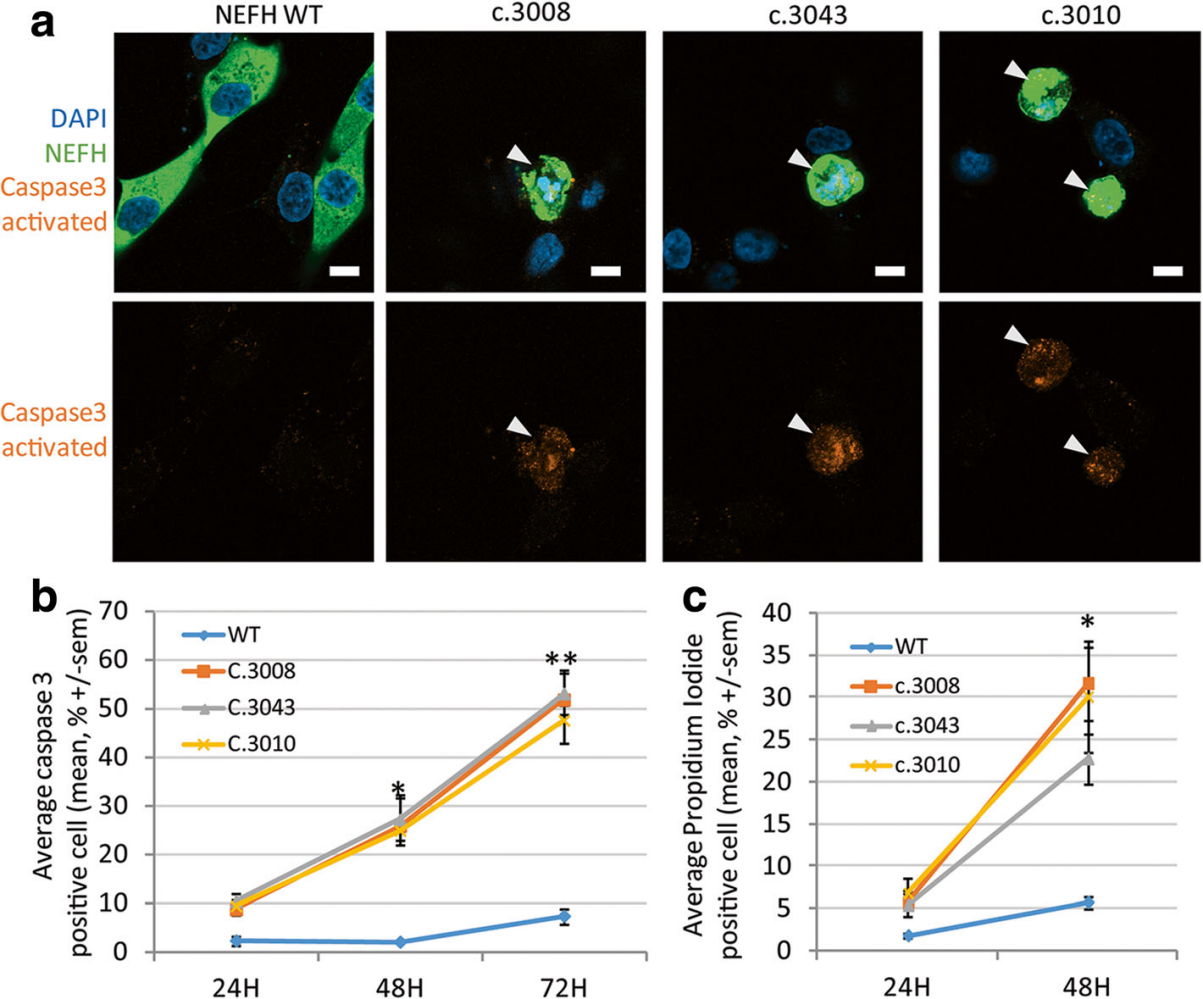
NEFH mutations trigger caspase 3 dependent death in vitro. **a** Confocal images of transfected SH-EP counterstained with DAPI in *blue* and activated caspase 3 in *red*. *Scale bar*: 20 μm. **b** Percentage of caspase 3 activated positive cells overtime. Values represent means +/− sem from at least three independent experiment analyzed by one way ANOVA followed by multiple comparisons versus control group (Dunnett’s Method) * *p* < 0.02; ** *p* < 0.001. **c** Quantification of the percentage of propidium iodide permeable cells. Values represent means +/− sem from at least three independent experiment analyzed by one way ANOVA followed by multiple comparisons versus control group (Dunnett’s Method) * *p* < 0.02

We next examined whether mutant NEFH proteins could alter cell viability. Caspase 3 is activated by proteolytic cleavage by both extrinsic (death ligand) and intrinsic (mitochondrial) apoptotic pathways. Immunofluorescence experiments detected caspase 3 activation in numerous cells 48 h after transfection with one of the mutant NEFH expression vectors. Caspase 3 activation was often associated with a pyknotic nuclei, indicating ongoing apoptosis (Fig. 5a). Quantification of the percentage of cells containing activated caspase 3 at different times points after transfection revealed a progressive increase of the number of cells triggering the apoptotic pathway. Approximately 10% of SH-EP cells expressing a mutant NEFH had activated caspase 3 twenty four hours after transfection, this number increasing to 25% and 50% 48 h and 72 h after transfection, respectively (Fig. 5b).

To confirm that mutant NEFH triggered cell death overtime, we used propidium iodide (PI), a fluorescent intercalating agent that requires broken membranes to reach nuclear DNA and thus selectively labels dying cells. Twenty-four hours after transfection with a mutant NEFH vector, 6% of cells were stained positive for PI, and 25% of cell were stained 1 day later, consistently with caspase 3 activation results (Fig. 5c). Altogether, these results show that expression of mutant NEFH strongly triggered caspase 3 activation and cell death.

### Mutant NEFH form aggresomes in primary motoneurons in vitro

**Fig. 6:**
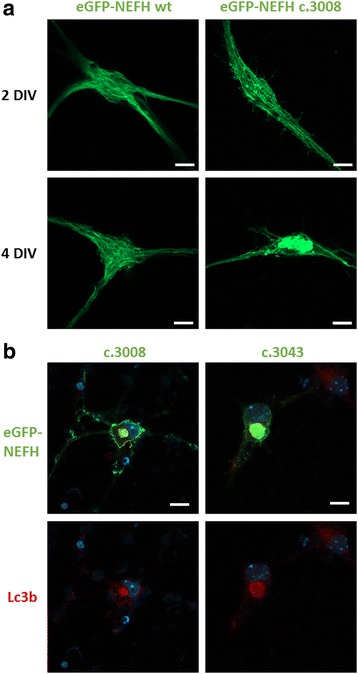
NEFH mutations form aggresome in primary motoneuron in vitro. **a** confocal images of mice primary motoneuron culture expressing eGFP-NEFH WT or c.3008 mutation 2 or 4 days in vitro (DIV) after magnetofection. Scale bar: 10 μm. **b** eGFP-NEFH mutant proteins form agresome after 4 DIV that colocolised with LC3b. *Scale bar*: 10 μm

To confirm our observation in motoneurons, mouse primary motoneuron cultures were used [14, 16, 17] (Additional file 4: Figure S4). Two days after magnetofection, WT and mutant eGFP-NEFH formed a filamentous network with endogenous NEFL (Fig. 6a). Interestingly, after 2 days of expression mutant eGFP-NEFH formed aggregates along the filamentous structure, which evolved in a prominent perinuclear aggresome containing LC3b as observed 4 days after magentofection (Fig. 6a and b).

### NEFH mutations cause protein aggregation and apoptosis in spinal cord neurons

**Fig. 7:**
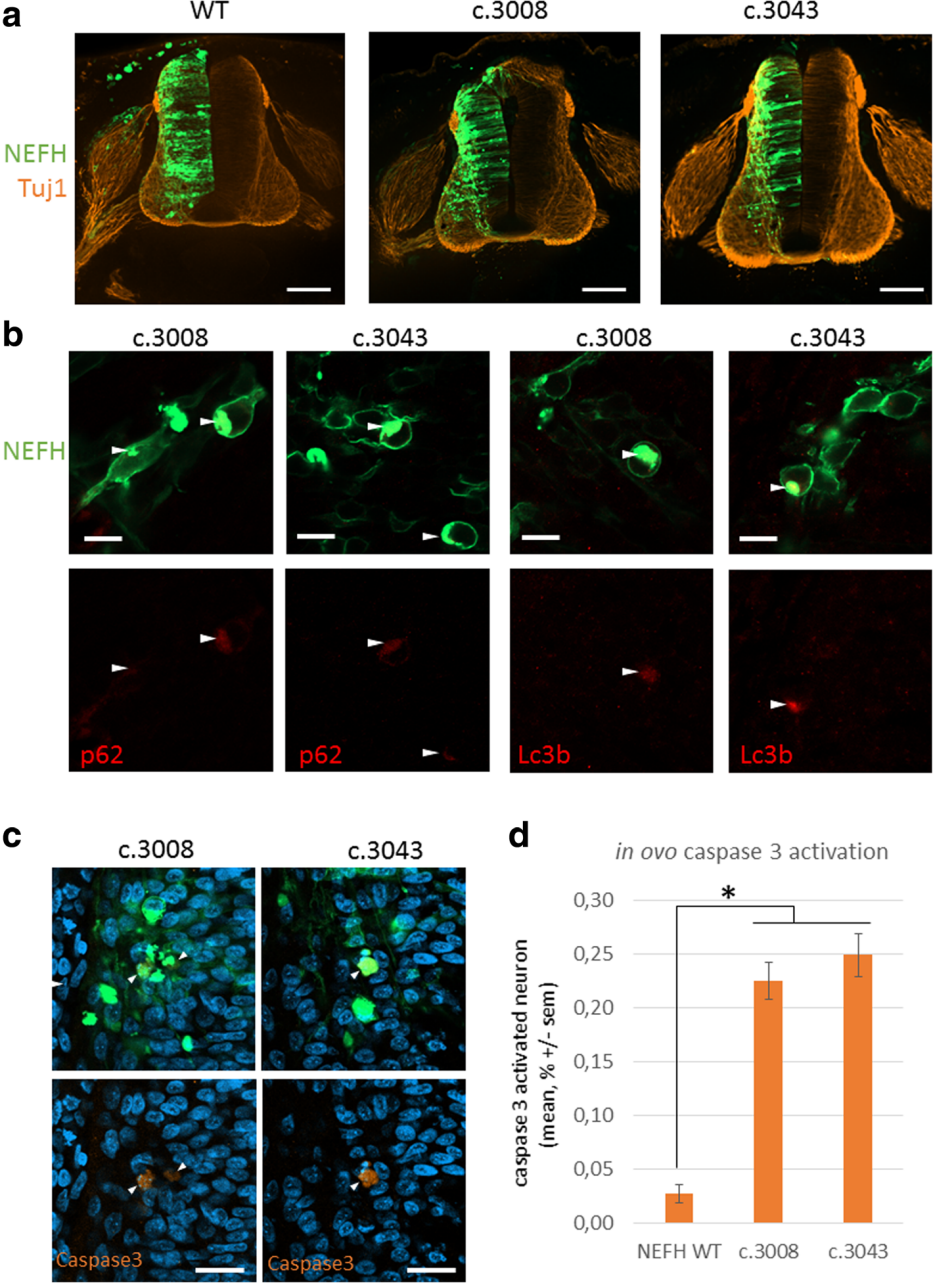
mutant NEFH aggregate in ovo and trigger apoptosis. **a** confocal images of chick embryo spinal cord cryosection after in ovo electroporation. Sections of 20 μm were counterstained for beta3-tubulin (TUJ1 clone), a neuronal specific isoform. Scale bar represent 100 μm. **b** Zoom of the anterior horn of the spinal cord to visualize mutant NEFH aggregates in the soma of electroporated neurons, which co localize with p62/SQSTM1 and LC3b. Scale bar: 10 μm. **c** Caspase 3 activated staining of the spinal cords appears in some neurons harboring mutant NEFH protein aggregates. Scale bar represent 20 μm. Arrow show apoptotic cells. **d** Quantification of caspase 3 activation in electroporated neurons. Values represent means of percentage +/− sem from at least 20 electroporated 20 μm thick cryosections of four different embryos per condition and analyzed by one way ANOVA followed by multiple comparisons versus control group (Dunnett’s Method) * *p* < 0.001

In order to evaluate the effect of NEFH mutations in vivo on spinal cord neurons, we decided to express NEFH in the spinal cord of chick embryos by in ovo electroporation. Electroporation of embryonic hemi neural tube allows transfer of expression vectors in both motor neurons and dorsal root ganglion (DRG) sensitive neurons. Confocal images of the entire spinal cord showed that transfection was efficient in the hemi spinal cord neurons (Fig. 7a) and less efficient in the DRG but still sufficient to visualize some electroporated neurons (data not show). At higher magnification the anterior horn of these sections holding motor neurons revealed that WT NEFH distributed in a filamentous network (Additional file 2: Figure S2C). Conversely, mutant NEFH proteins accumulated at intense foci in the soma of the neurons forming aggresomes which colocalized with p62/SQSTM1 and LC3b (Fig. 6b). Caspase 3 activation was clearly detected in 0.22% +/−0.02 and 0.25% +/−0.03 neurons electroporated with mutant NEFH constructs c.3008 and c.3043 respectively (Fig. 7c). In these neurons, the occurrence of Caspase 3 activation 48 h after electroporation was more than 8 fold higher than in neurons expressing the WT NEFH expression construct (Fig. 7d). Progressive apoptosis activation is consistent with the neurodegenerative features observed in the patients.

## Discussion and conclusions

The families presented here displayed a severe, predominantly motor, axonal autosomal dominant form of CMT with proximal motor involvement in the lower limb associated with two previously unreported mutations in the *NEFH* gene, including a de novo mutation. The clinical phenotype varied in term of age at onset, ranging from 5 to 40 years, with a median around 30 years. A remarkable and consistent feature in all patients was the early involvement of proximal muscles of the lower limbs, occurring approximately 10 to 15 years after the onset of motor deficit. Proximal deficit involved predominantly the iliopsoas muscle, whereas quadriceps and hamstring muscles were relatively preserved. Proximal muscles weakness is rare in any form of CMT. Proximal lower-limb weakness with waddling gait is also described in recessive forms of CMT due to *NEFL* mutations [2]. There was muscle wasting predominant in the distal lower limbs muscles progressively ascending to proximal limbs muscles and distal upper limbs muscles. Muscle weakness and muscle wasting were quickly evolving, with most of the patients needing walking assistance after 20 years of disease evolution. Three patients in family 1 had brisk reflexes associated with distal and proximal weakness. Pyramidal signs have also been shown in CMT caused by *NEFL* mutations [2, 13]. The phenotype was mainly motor but there were also sensory alterations, with prominent involvement of large sensory nerves. There were no associated features except for gastroparesia in one patient and vocal cord involvement in another patient. Muscle pathology and electrophysiological studies were consistent with a symmetrical, progressive distal and proximal sensorimotor axonal neuropathy. These findings are in accordance with the previously published paper [27]. It is worth noticing that electrophysiological studies of two asymptomatic patients of family 1 (IV5, IV6) showed a sensorimotor neuropathy respectively at the age of 23 and 17 years, indicating that the neuropathy may exist in a latent state. Patients of these IV generation have been diagnosed early in their life since they were aware of symptoms related to the neuropathy in the family. Although we could not exclude the hypothesis of an anticipation, it must be stressed that even mild neurological symptoms led their parents to seek for a neurological and neurophysiological exam that would confirm the neuropathy.

The pathogenicity of the *NEFH* mutations was confirmed by the easy segregation of the mutation in both families. The clinical phenotype was also very similar in the two families. Analysis of family 1 suggests an anticipation pattern given that the proband had an earlier age of onset and more severe manifestations than did the previous generations. This anticipation pattern has already been reported in the previously reported patients [27]. Nevertheless, this anticipation phenotype is probably rather due to a diagnostic bias than to biological phenomenon. Indeed, in families with known familial CMT, clinical signs of the disease are searched earlier and thus detected sooner in the offsprings. The penetrance of the disease appeared to be complete. Muscle weakness was more severe in patient II-1 of family 2, who carries a de novo mutation in *NEFH* gene, than in patients belonging to family 1 at the same age. The two previously reported families had frameshift variants in the extreme C-terminus of NEFH [27]. Our two families present with original deletions of two nucleotides close to the normal stop codon. Mutation in family 1 (c.3008_3009delAA; p.Lys1003Argfs*59) leads to the same defective protein as in family UK1 at the exception of one amino acid. Mutation in family 2 is located just three amino acids before the normal stop codon, and is the most subterminal variant in the four families reported thus far. Both mutations translate into the same alternative Open Reading Frame (ORF), which is also identical to the previously reported families [27].

The predominance of motor involvement in both proximal and distal muscles associated with brisk reflexes in some members of family 1 initially prompted us to study the *SOD1* gene implicated in genetic ALS, but no mutation was found. In ALS, there is accumulation of neurofilaments in motor neurons. Mouse models with overexpression of neurofilaments subunits have a motor neuropathy resembling ALS [20]. Rare mutations in the peripherin and in the KSP repeats motifs have been reported [1, 8], and a latter study showed that a short allele of the *NEFH* tail was associated with ALS [30]. Other studies found no mutations of *NEFH* in ALS [9, 28]. The pathogenicity of *NEFH* mutations in ALS thus remains unclear. Nevertheless, the proposed association of *NEFH* mutations with ALS and the finding that some CMT patients with *NEFH* mutations have unusual clinical signs related to ALS is intriguing and consistent with the notion that mutations causing the addition of a cryptic amyloidogenic element to NEFH proteins cause a particular neuropathy with overlapping clinical features of both CMT and ALS.

Our in vitro and in vivo experiments provide a rational basis to these observations by demonstrating that *NEFH* mutations causing the addition of a CAE induce aggresome formation and neuronal apoptosis. Loss of motor neurons is a hallmark of ALS, which is also increasingly associated with the presence of protein aggregates in motoneurons. This is especially true for ALS caused by mutations in *SOD1*, *TDP43* and *C9ORF72* [32]. The presence of intracellular protein inclusions is a common hallmark of a wide variety of human disorders. These include neurofibrillary tangles in Alzheimer’s disease, Lewy bodies in Parkinson’s disease, polyglutamine enriched inclusions in Huntington’s disease, as well as intermediate filament inclusions in specific myopathies. Once formed, protein aggregates tend to be insoluble, refractory to proteolysis and to accumulate in inclusion bodies which are usually present in low copy number, most often only one per cell [18]. This is consistent with the features of the NEFH protein aggregates we observed, which mostly accumulated in one spot and contained insoluble proteins.

Autophagy is the major cellular process by which large cytoplasmic components, including ribosomes, organelles and protein aggregates, are degraded. Autophagy plays a key role in the maintenance of homeostasis and the quality of the cellular component for the survival of the neuron in their functional context. The finding that the autophagy proteins p62 and LC3 are present in NEFH aggregates indicates that the presence of the CAE triggers autophagy. Although the precise role of autophagy in motoneuron disease is unclear, emerging evidence supports the notion that defects in autophagic pathway may contribute to pathogenic mechanism and could constitute valuable therapeutic targets [5, 7, 22, 31].

Mutations in *NEFH* cause a sensorimotor axonal neuropathy, characterized by distal lower limbs motor deficit with early and prominent involvement proximal of the iliopsoas muscle, associated in some patients with pyramidal signs. Clinical features of *NEFH* mutations clearly overlap with those of motor neuron disease. At the cellular level, mutant NEFH is sequestered in a prominent perinuclear inclusion body, the aggresome which is addressed to the autophagic pathway. Mutant NEFH proteins are nevertheless toxic and progressively trigger caspase 3 dependant apoptosis. Interestingly, motoneuron death and intracellular protein inclusions are common hallmarks in ALS. Characterization of cellular effects of the *NEFH* mutations provides a rational basis to the clinical continuum between CMT and ALS in affected patients.

## Additional files

Additional file 1: Figure S1. Superficial peroneal sensory nerve biopsy (case III4): Semi-thin section. A. Note the rarefaction of large myelinated fibers. B. Several fibers have a thin myelin sheath (arrowhead) and some of them present myelin sweling (arrow). (Original magnification ×100). (PNG 947 kb)

Additional file 2: Figure S2. eGFP-NEFH WT form filamentous network in vitro and in ovo. A. monomeric eGFP tag NEFH WT expression can form visible filamentous network in SH-EP under lower expression condition when transfected at low concentration (optimal recommended concentration diluted four time). eGFP-NEFH WT form filamentous network in spinal motoneuron in vitro (B) and in vivo (C). Scale bar 10 μm. (TIFF 156 kb)

Additional file 3: Figure S3. NEFH mutations modify cell morphology in vitro*.* A Mutant NEFH expression induces morphological changes as seen on 10× microscopic images. Scale bar represent 100 μm. B-C. Quantification of the average shape factor and radius of transfected SH-EP cells. Values represent means in percent +/− standard deviation of at least 15 fields (Cells analyzed >1000 per condition) and analyzed by Kruskal-Wallis one way ANOVA on ranks test followed by Dunn’s methods (*P < 0.001). Shape factor equal to one usually represents an ideal circle. (PNG 334 kb)

Additional file 4: Figure S4. Representative primary motoneuron in its entirety in vitro. A Non transfected motoneuron revealed by SMI-32 staining. B. Magnetofected motoneuron with eGFP tag NEFH WT or mutated form, without counterstaining. (PNG 156 kb)
