# Supplementary figures and images for: Cryptic amyloidogenic elements in mutant NEFH causing Charcot-Marie-Tooth 2 trigger aggresome formation and neuronal death
Source: Acta Neuropathol Commun. 2017 Jul 14;5:55. doi: 10.1186/s40478-017-0457-1 (PMC5513089; doi:10.1186/s40478-017-0457-1)

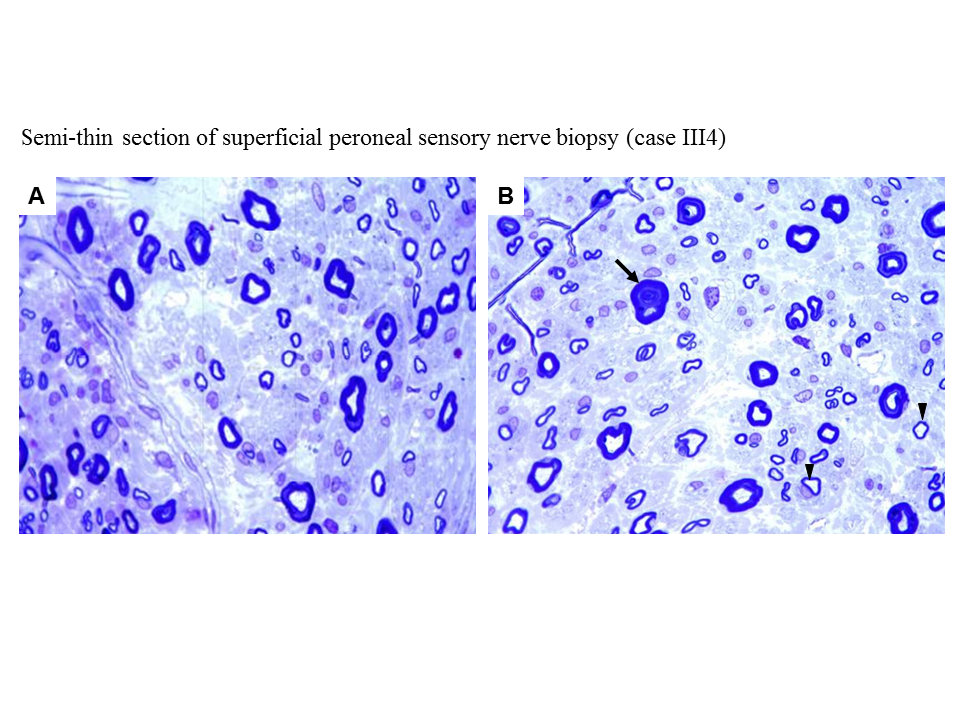

Supplement: Supplementary file 1 — Superficial peroneal sensory nerve biopsy (case III4): Semi-thin section. A. Note the rarefaction of large myelinated fibers. B. Several fibers have a thin myelin sheath (arrowhead) and some of them present myelin sweling (arrow). (Original magnification ×100). (PNG 947 kb) [file 40478_2017_457_MOESM1_ESM.png]

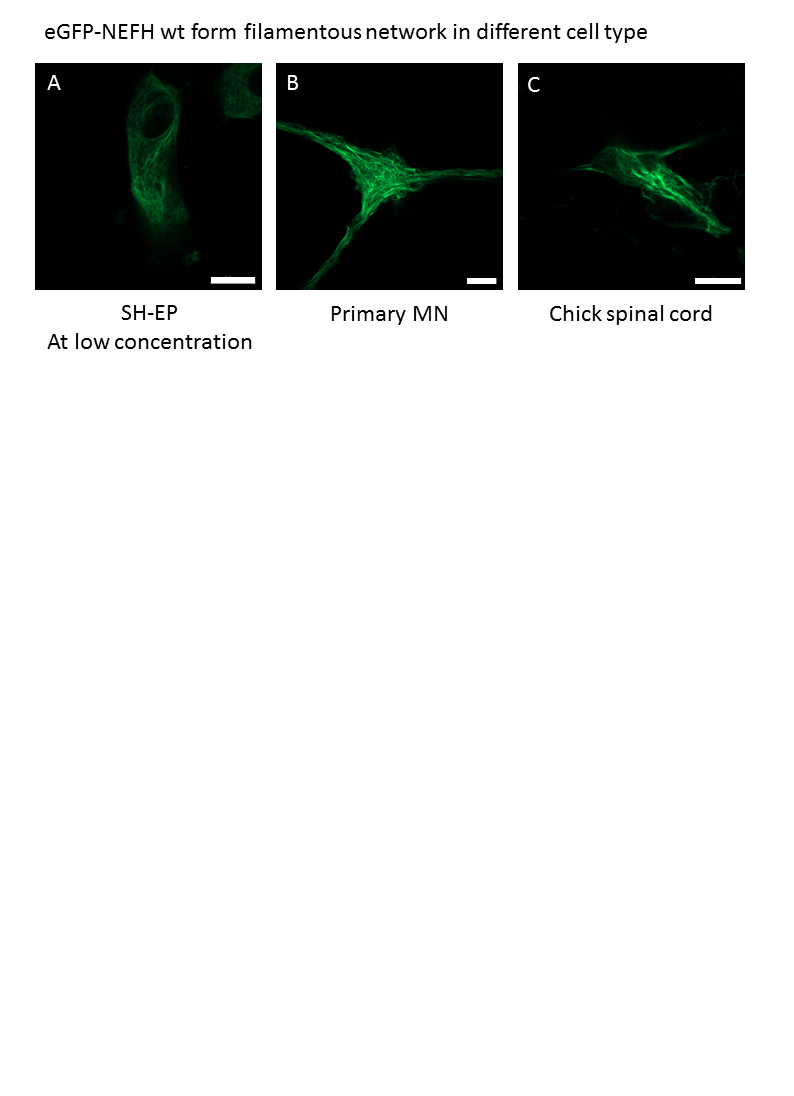

Supplement: Supplementary file 2 — eGFP-NEFH WT form filamentous network in vitro and in ovo. A. monomeric eGFP tag NEFH WT expression can form visible filamentous network in SH-EP under lower expression condition when transfected at low concentration (optimal recommended concentration diluted four time). eGFP-NEFH WT form filamentous network in spinal motoneuron in vitro (B) and in vivo (C). Scale bar 10 μm. (TIFF 156 kb) [file 40478_2017_457_MOESM2_ESM.tif]

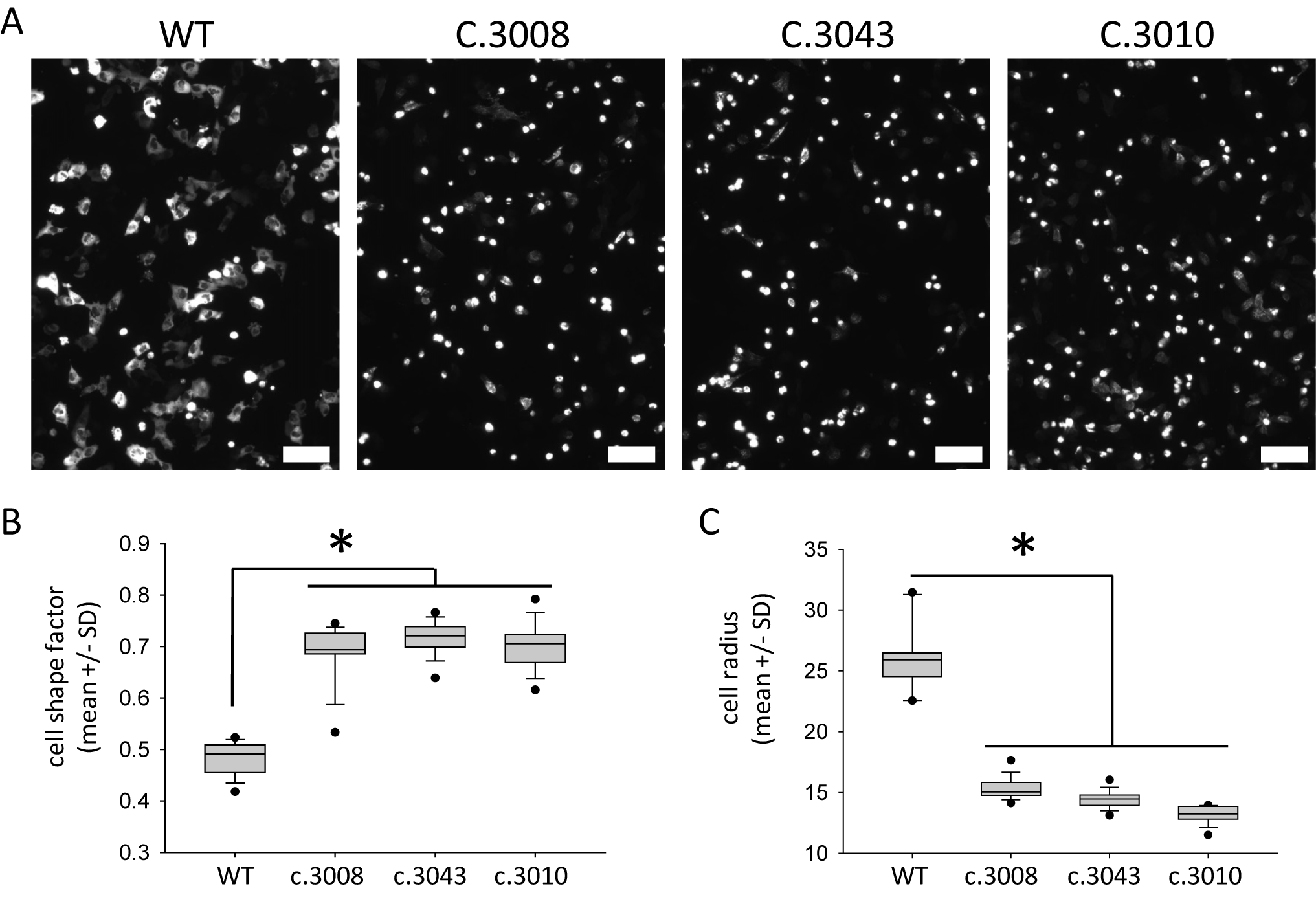

Supplement: Supplementary file 3 — NEFH mutations modify cell morphology in vitro. A Mutant NEFH expression induces morphological changes as seen on 10× microscopic images. Scale bar represent 100 μm. B-C. Quantification of the average shape factor and radius of transfected SH-EP cells. Values represent means in percent +/− standard deviation of at least 15 fields (Cells analyzed >1000 per condition) and analyzed by Kruskal-Wallis one way ANOVA on ranks test followed by Dunn’s methods (*P < 0.001). Shape factor equal to one usually represents an ideal circle. (PNG 334 kb) [file 40478_2017_457_MOESM3_ESM.png]

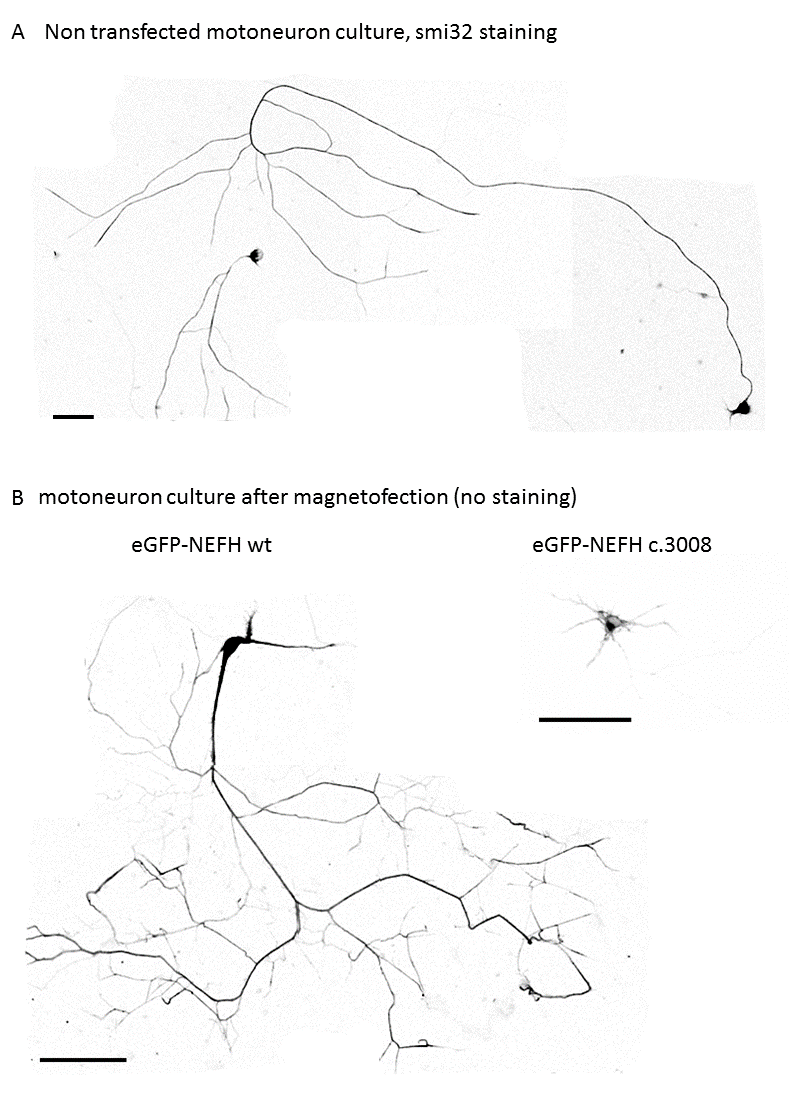

Supplement: Supplementary file 4 — Representative primary motoneuron in its entirety in vitro. A Non transfected motoneuron revealed by SMI-32 staining. B. Magnetofected motoneuron with eGFP tag NEFH WT or mutated form, without counterstaining. (PNG 156 kb) [file 40478_2017_457_MOESM4_ESM.png]
